# Increasing Sodium Variability in the First 96 Hours after Birth is Associated with Adverse In-Hospital Outcomes of Preterm Newborns

**DOI:** 10.1016/j.cdnut.2022.100026

**Published:** 2022-12-27

**Authors:** Olivia C. Brandon, Krystle M. Perez, Sarah E. Kolnik, Sandra E. Juul, Thomas R. Wood, Gregory C. Valentine

**Affiliations:** 1 Division of Neonatology, University of Washington/Seattle Children’s Hospital, Seattle, WA, USA; 2 Center on Human Development and Disability, University of Washington, Seattle, WA, USA; 3 Department of Obstetrics & Gynecology at Baylor College of Medicine, Houston, TX, USA

**Keywords:** sodium, osmolality, variability, mortality, necrotizing enterocolitis, prematurity, neonatology

## Abstract

**Background:**

Neonatal intraventricular hemorrhage prevention bundles for preterm infants commonly defer daily weighing for the first 72 h, with reweighing occurring on day 4. Clinicians rely on maintaining stable sodium values as a proxy of fluid status to inform fluid management decisions over the first 96 h after birth. Yet, there exists a paucity of research evaluating whether serum sodium or osmolality are appropriate proxies for weight loss and whether increasing variability in sodium or osmolality during this early transitional period is associated with adverse in-hospital outcomes.

**Objectives:**

To evaluate whether serum sodium or osmolality change in the first 96 h after birth was associated with percent weight change from birth weight, and to assess potential associations between serum sodium and osmolality variability with in-hospital outcomes.

**Methods:**

This retrospective, cross-sectional study included neonates born at ≤30 gestational weeks or ≤1250 g. We evaluated associations between serum sodium coefficient of variation (CoV), osmolality CoV, and maximal weight loss percentage in the first 96 h after birth with in-hospital neonatal outcomes.

**Results:**

Among 205 infants, serum sodium and osmolality were poorly correlated with percent weight change in individual 24-h increments (*R*^*2*^ = 0.01–0.14). For every 1% increase in sodium CoV, there was an associated 2-fold increased odds of surgical necrotizing enterocolitis and 2-fold increased odds of in-hospital mortality (odds ratio, 2.07; 95% CI: 1.02, 4.54; odds ratio, 1.95; 95% CI: 1.10, 3.64, respectively). Sodium CoV was more strongly associated with outcomes than absolute sodium maximal change.

**Conclusions:**

In the first 96 h, serum sodium and osmolality are poor proxies for assessing percent weight change. Increasing variability of serum sodium is associated with later development of surgical necrotizing enterocolitis and all-cause in-hospital mortality. Prospective research is needed to evaluate whether reducing sodium variability in the first 96 h after birth, as assessed by CoV, improves newborn health outcomes.

## Introduction

Intraventricular hemorrhage (IVH) remains an important and potentially preventable cause of long-term neurocognitive morbidity in preterm infants. Strategies to reduce the risk of IVH during the most vulnerable 72 h after birth frequently include midline positioning and minimizing handling, including deferring daily weights [1]. With deferred daily weighing, infants are weighed at birth and then the next weight is typically obtained on day 4. As weight change over the first days after birth is associated with overall fluid balance as well as in-hospital outcomes, clinicians caring for newborns commonly use weight change as a key indicator of overall fluid status and well-being [[2], [3], [4], [5], [6], [7], [8], [9], [10], [11]]. Without daily weights in the first 72 to 96 h after birth, clinicians must rely on presumed proxies such as serum sodium or serum osmolality to inform their fluid management decisions during this critical transitional period.

Although correlation between serum sodium and weight loss has been established for older children and adults, complex fluid and electrolyte shifts occur as preterm infants transition from fetal-to-neonatal physiology, and these shifts may impact the reliability of electrolytes as proxies for hydration status [5,[12], [13], [14], [15], [16]]. Moreover, the long-term consequences of variability in serum sodium and osmolality on preterm outcomes are poorly understood.

Studies have demonstrated an association between *absolute* change in serum sodium (the difference between sodium at 2 specific time points) and IVH [17]. The coefficient of variation (CoV), the ratio of the standard deviation to the mean value, incorporates multiple values over a period of time and therefore provides a potentially more accurate measure of variability. No studies have evaluated the impact of increased variability of serum sodium or osmolality in the first 4 d after birth on neonatal in-hospital morbidity or all-cause mortality. Thus, key questions remain: *1*) is serum sodium a reasonable proxy for weight loss in the first 96 h after birth, and *2*) which correlates best with adverse health outcomes, absolute change or CoV of serum sodium values? The University of Washington Neonatal Intensive Care Unit (NICU) implemented an IVH Prevention Bundle in 2018 that first included daily weights, followed by a period in which daily weights were not obtained for the first 3 d after birth. Using a retrospective cross-sectional analysis of this population, we now address these 2 questions.

## Methods

A retrospective cross-sectional study was performed including 205 neonates born ≤1250 g or ≤30 wk gestation and admitted to the University of Washington Level IV NICU in Seattle, Washington between February 8, 2018 and March 19, 2021. Infants with congenital anomalies, diagnosed genetic conditions, and those who did not survive the first 96 h after birth were excluded from all analyses. Institutional Review Board approval was received from the University of Washington (Study 00006091). The study was performed in accordance with the Declaration of Helsinki (revised 2013).

An IVH Prevention Bundle was implemented in 2 phases, the first with and the second without daily weighing. From February 8, 2018 to October 31, 2019, the bundle included daily weights in the first 72 h after birth; between November 1, 2019 and March 19, 2021, the IVH Prevention Bundle did not permit weighing until 96 h after birth. To determine the association between percent weight change and serum osmolality or serum sodium, data from all neonates with daily weight measurements and serum biomarkers recorded on days 1 through 4 after birth were utilized (*n* = 109). The percent weight change and change in serum sodium and osmolality were measured daily.

We calculated the CoV in serum osmolality and serum sodium as percent CoV ($\frac{standard\;deviation}{mean}\times 100$) over the first 96 h after birth and assessed their association with in-hospital outcomes. Serum osmolality was calculated using $2\times \left[Na\right]+\frac{\left[BUN\right]}{2.8}+\frac{\left[Glucose\right]}{18}$. The first daily serum sodium (mEq/L) and blood urea nitrogen (BUN) values were used, and the average of the daily highest and lowest glucose (mg/dL) was used in the CoV calculation. We calculated the absolute maximum sodium change as the difference between the highest and lowest recorded serum sodium values within the first 96 h after birth. We defined day 0 as the day of birth. The primary outcomes analyzed were IVH (Papile grade I–IV, either unilateral or bilateral) [18], bronchopulmonary dysplasia (BPD, oxygen requirement at 36 wk postmenstrual age) [19], any necrotizing enterocolitis (NEC, defined using modified Bell’s staging IIA or higher) [20,21], NEC requiring surgical treatment, patent ductus arteriosus (PDA) requiring surgical treatment, in-hospital mortality, and length of hospital stay.

Infants diagnosed with IVH within the first 96 h after birth were excluded from analyses assessing IVH as an outcome as our current unit protocol is to obtain the first cranial ultrasound at 7–10 d after birth; any cranial ultrasound performed earlier was per individual clinician’s decision. Common reasons for doing off-protocol early head ultrasounds at our institution include, but are not limited to: severe hypotension, unexplained acute anemia, increasing vasopressor requirements, or to guide discussions about redirection to comfort care; thus, we reasoned this was a sicker, atypical group and they were excluded. Infants who did not live long enough to be diagnosed with clinical outcomes of interest were excluded from that specific analysis. Thus, infants who did not survive 1 week after birth were excluded from IVH (due to lack of cranial ultrasound obtained at 7–10 d after birth) and NEC analyses; infants who did not survive to 36 wk postmenstrual age were excluded from PDA and BPD analyses. Only infants who were discharged home were included in analyses examining length of stay (Supplemental Figure 1).

At our institution, it is protocolized to provide minimal amounts of sodium-containing fluids within the first 48–72 h after birth. Starting on day 2, 1–2 mEq/kg of sodium is provided via parenteral nutrition. Umbilical arterial lines commonly use sodium acetate and are routinely used in infants <28 wk gestation, starting after birth. No other parenteral or enteral supplementation is provided during this period.

### Statistical analyses

Demographic variables for those exposed to IVH bundles with and without daily weighing were compared by Fisher’s exact tests and Mann-Whitney *U* tests for categorical and continuous variables, respectively. Linear regression models were used to predict percent weight change for each 24-h period during the first 96 h after birth, using either change in serum sodium or serum osmolality as a predictor after adjusting for gestational age (GA), small-for-gestational-age (SGA) status (using Fenton growth curves with SGA defined as <10% birth weight for gestational age) [22], vasopressor use in first week after birth, daily urine output (mL/kg birth weight/h), daily fluid amount (mL/kg birth weight/d), and highest daily glucose (mg/dL).

To control for potential known confounders, we utilized logistic regression models to predict categorical in-hospital outcomes using either serum sodium CoV, osmolality CoV or absolute maximal sodium change over the first 96 h after birth. Models were adjusted for the following potential confounders: GA, whether the infant was weighed in the first 0–72 h, SGA status, vasopressor use in the first week after birth, average daily urine output within 96 h after birth, average daily fluid intake within 96 h after birth, weight loss <5% within 96 h after birth, weight loss >15% within 96 h after birth, lowest sodium <135 mEq/L or lowest osmolality <280 mEq/L within 96 h after birth, highest sodium >145 mEq/L or highest osmolality >300 mEq/L within 96 h after birth, and highest glucose within 96 h after birth [[23], [24], [25]]. Glucose was not included in the osmolality model as it was used in the osmolality calculation.

These variables were selected because of their known association with alterations in serum sodium values, GA [26], SGA [27], urine output [5], daily fluid intake [28], and glucose values [29] or likelihood of reflecting higher severity of illness (i.e., vasopressor use). Previous research has shown associations of neonatal outcomes and sex, with male infants having higher rates of mortality and respiratory comorbidities; therefore, we included sex as a potential confounder [30]. Additionally, our previous work evaluating extremely preterm newborns demonstrated maximal weight loss from birthweight categories of <5%, 5%–15%, or >15% had differing associations with in-hospital outcomes [11]. Finally, in an effort to account solely for the variability of serum sodium or osmolality on the in-hospital outcomes rather than the degree of hypo- or hypernatremia (or osmolality), we corrected for these low or high values in our model [[23], [24], [25]].

Linear regression models evaluated the association of the *1*) serum sodium CoV, *2*) osmolality CoV, and *3*) absolute maximal sodium change with the length of hospital stay after adjusting for GA, daily weighing, SGA status, vasopressor use in first week after birth, average urine output within 96 h after birth, average fluid intake within 96 h after birth, weight loss <5% 0–96 h after birth, weight loss >15% 0–96 h after birth, lowest sodium <135 mEq/L or lowest osmolality <280 mEq/L within 96 h after birth, highest sodium >145 mEq/L or highest osmolality >300 mEq/L within 96 h after birth, and highest glucose in the first 96 h after birth.

To investigate the optimal thresholds for absolute maximal sodium change and sodium CoV, the cutpointr library in R was used to identify thresholds that provided the highest sum of sensitivity and specificity to predict mortality [31]. These thresholds were then used to convert the continuous variables of absolute maximal sodium change and sodium CoV into categorical variables. Logistic regression using the thresholds for absolute maximal sodium change and sodium CoV was utilized to predict categorical in-hospital outcomes, including adjustments for the same potential confounders as described above.

Statistical analyses were performed in Prism version 9.2.0 (GraphPad Software) and R version 4.1.1 (R Foundation for Statistical Computing) [32]. A probability (*P*) level of less than 0.05 was considered significant.

## Results

Infants included in the IVH Prevention Bundle with (*n* = 109) and without (*n* = 96) daily weights are presented in Table 1. Infants in the bundle with daily weights were of younger GA, more likely to require vasopressors, and had higher average urine output over the first 96 h after birth. These differences were included in all adjusted models.

**TABLE 1:** Infant characteristics

	IVH bundle with weighing *N* = 109	IVH bundle without weighing *N* = 96	*P*
	*n*/*N* (% of data) / median (IQR)	*n*/*N* (% of data) / median (IQR)	
Gestational age (wk)	26.7 (25.0, 28.6)	27.7 (26.5, 29.3)	*0.005*
Birth weight (g)	871.0 (678.0, 1127)	966.0 (736.3, 1128)	0.20
Percent weight loss from birth weight at day 4	8.0 (3.9, 12.4)	8.7 (3.7, 12.5)	0.97
Female sex	41/109 (37.6)	39/96 (40.6)	0.67
Received vasopressors in first week after birth	30/109 (27.5)	12/96 (12.5)	*0.009*
SGA	20/109 (18.3)	22/96 (22.9)	0.49
Average total fluid intake in first 96 h after birth (mL/kg/d)	141.6 (131.6 152.4)	137.1 (129.8, 146.0)	0.06
Average urine output in first 96 h after birth (mL/kg/h)	3.8 (3.5, 4.5)	3.6 (3.2, 4.1)	*0.002*
Sodium coefficient of variation in first 96 h after birth >3%	40/109 (36.7)	31/96 (32.3)	0.56
Absolute maximal change in sodium in first 96 h after birth ≥9 mEq/L	50/109 (45.9)	38/96 (39.6)	0.40
IVH	21/94 (22.3)	22/90 (24.4)	0.86
All NEC	11/106 (10.4)	5/96 (5.2)	0.20
Surgical NEC	9/106 (8.5)	2/96 (2.1)	0.06
Surgical PDA	10/98 (10.2)	6/88 (6.8)	0.45
BPD	55/98 (56.7)	39/88 (45.3)	0.14
Mortality	12/109 (11.0)	8/96 (8.3)	0.64
Length of hospital stay (d)	89.0 (65.5, 119.3)	82.0 (66.0, 100.0)	0.35

Infant characteristics for the cohorts on the IVH Prevention Bundle with daily weighing and on the IVH Prevention Bundle without daily weighing. Mann-Whitney *U* test or Fisher’s Exact Test revealed differences in gestational age, vasopressor use, and average urine output between the groups. Significant *P* values italicized. BPD, bronchopulmonary dysplasia; IQR, interquartile range; IVH, intraventricular hemorrhage; NEC, necrotizing enterocolitis; PDA, patent ductus arteriosus; SGA, small for gestational age.

### Association between serum sodium and osmolality values with percent weight change from birth weight

**FIGURE 1:**
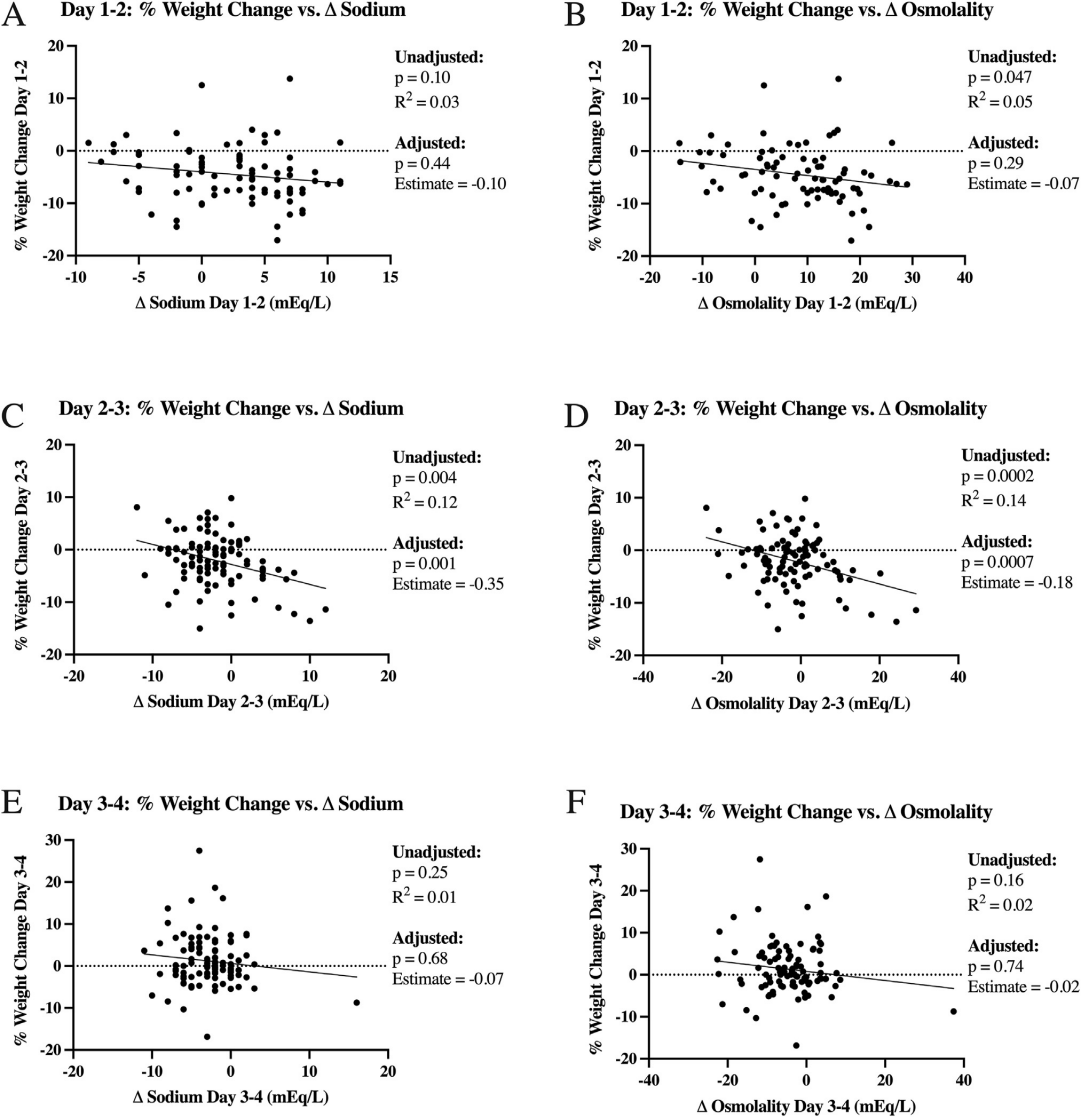
**Serum sodium, osmolality, and weight change for each 24-h increment**. Unadjusted regressions of serum sodium or serum osmolality and associated weight change over 24 h among *n* = 109 neonates who received daily weights 0–72 h after birth. Unadjusted linear regressions for day 1 to day 2 change in serum sodium (A) or serum osmolality (B), day 2 to day 3 change in serum sodium (C) or serum osmolality (D), and day 3 to day 4 change in serum sodium (E) or serum osmolality (F) with weight change. Adjusted models included total fluid intake (mL/kg/d), urine output (mL/kg/h), gestational age, vasopressor use in the first week, small-for-gestational-age status, and maximum glucose (mg/dL) from the day before electrolytes were measured. Glucose was not included in the osmolality model as it was used in the osmolality calculation. Only serum electrolytes day 2 to day 3 predicted weight change in unadjusted and adjusted regressions, but the effect was small.

The percent weight change associated with change in serum sodium and osmolality for sequential 24-h intervals after birth is shown in Figure 1. Unadjusted and adjusted models are indicated, with adjustments including fluid intake in the preceding 24-h period, urine output in the preceding 24-h period, GA, vasopressor use in the first week after birth, SGA, and maximal glucose in the preceding 24-h period. Change in serum sodium was only significantly associated with percent weight change from day 2 to day 3 after birth, but the effect was minimal; for every increase of 1 mEq/L of serum sodium, there was an associated increase in percent weight loss by 0.35% in the adjusted model [estimate, -0.35; 95% CI: -0.55, -0.14], with change in serum sodium only predicting 12% of the variance in weight change in unadjusted models (*R*^*2*^ = 0.12). Urine output was significantly associated with percent weight change from day 2 to day 3 (estimate, -0.76; 95% CI: -1.45, -0.08). Fluid intake, urine output, and maximum glucose were significantly associated with percent weight change from day 3 to day 4 after birth (estimate, 0.11; 95% CI: 0.04, 0.17; estimate, -1.42; 95% CI: -2.37, -0.46; estimate, 0.02; 95% CI: 0.0004, 0.04, respectively). GA, vasopressor use, and SGA were not significantly associated with percent weight change in any 24-h period.

Similar to changes in serum sodium, changes in serum osmolality were only significantly associated with percent weight change on day 2 to 3. For every increase of 1 mEq/L of serum osmolality, there was an associated increase in percent weight loss by 0.18% in the adjusted model (estimate, -0.18; 95% CI: -0.28, -0.08; *R*^*2*^ = 0.14). Associations between the other variables and percent weight change in the osmolality models were similar to those seen in the sodium models.

### Sodium and osmolality CoVs and association with in-hospital outcomes

**FIGURE 2:**
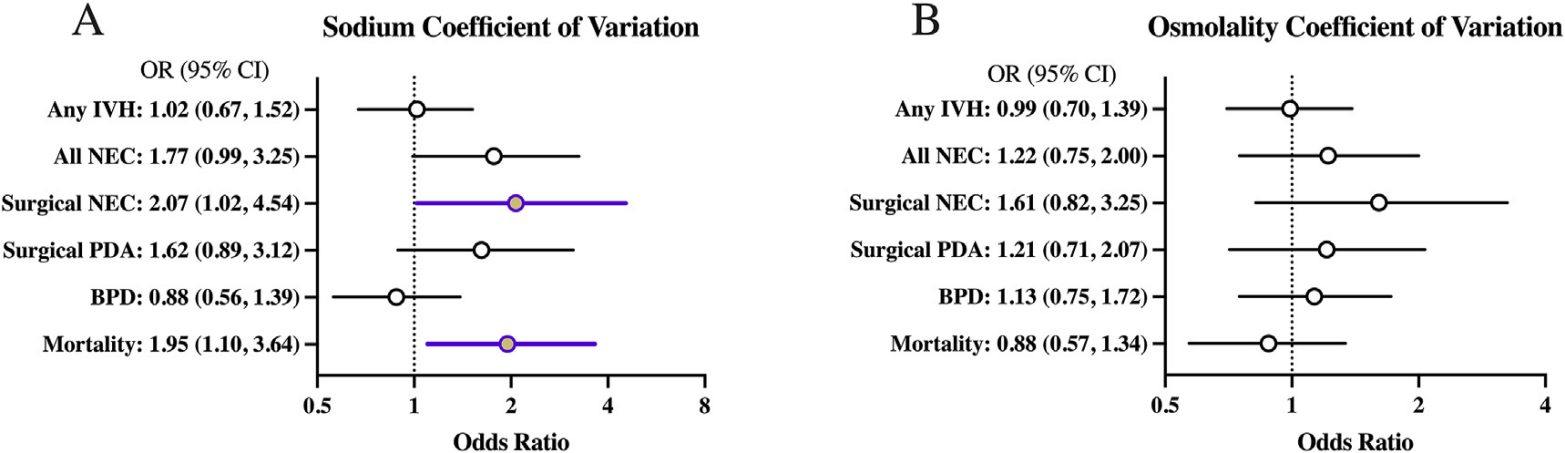
**Forest plots of serum sodium and osmolality coefficient of variation and association with in-hospital neonatal outcomes**. Logistic regression model predicting long-term outcomes using sodium coefficient of variation (A) or osmolality coefficient of variation (B) over the first 24–96 h after birth. Models were adjusted for gestational age, weighing, small-for-gestational-age (SGA) status, sex, vasopressor use in first week after birth, average urine output within 96 h after birth (mL/kg/h), average fluid intake within 96 h after birth (mL/kg/d), <5% weight loss 0–96 h after birth, >15% weight loss 0–96 h after birth, lowest sodium <135 mEq/L or lowest osmolality <280 mEq/L within 96 h after birth, highest sodium >145 mEq/L or highest osmolality >300 mEq/L, within 96 h after birth, and highest glucose within 96 h after birth (mg/dL). Glucose was not included in the osmolality model as it was used in the osmolality calculation. Odds ratios with 95% CIs listed for associations. Significant associations are shown in color. BPD, bronchopulmonary dysplasia; IVH, intraventricular hemorrhage; NEC, necrotizing enterocolitis; PDA, patent ductus arteriosus.

The association of each 1% increase in serum sodium CoV with specified clinical outcomes is shown in Figure 2A. Adjusted odds ratios (aOR) with 95% CIs are shown for each clinical outcome. Increased sodium CoV was significantly associated with increased odds of NEC requiring surgical treatment (aOR, 2.07; 95% CI: 1.02, 4.54) and in-hospital mortality (aOR, 1.95; 95% CI: 1.10, 3.64). Sodium CoV was not significantly associated with IVH, BPD, PDA requiring surgical treatment, or length of hospital stay (estimate of -1.74 for length of hospital stay; 95% CI: -5.79, 2.31; Figure 2A). The associations of serum osmolality CoV and the same clinical outcomes are shown in Figure 2B, none of which were statistically significant (estimate of -0.16 for length of hospital stay; 95% CI: -3.69, 3.37; Figure 2B). Neither weight loss <5% or >15% at 96 h compared with birth weight were associated with increased odds of any of the neonatal outcomes using either the sodium CoV model or the osmolality CoV model (TABLE 2, TABLE 3). Because newborns who lost >15% weight from birth weight at 96 h had higher sodium and osmolality CoV than the 5%–15% weight loss group (Supplemental Figure 2), we adjusted for weight loss groups in the adjusted models.

**TABLE 2:** Sodium model summary for in-hospital neonatal outcomes

	Any IVH	All NEC	Surgical NEC	PDA requiring surgical treatment	BPD	In-hospital mortality	Length of hospital stay (d)∗
Sodium coefficient of variation	1.02	1.77	*2.07*	1.62	0.88	*1.95*	-1.74
	(0.67, 1.52)	(0.99, 3.25)	*(1.02, 4.54)*	(0.89, 3.12)	(0.56, 1.39)	*(1.10, 3.64)*	(-5.79, 2.31)
Weight loss <5%	0.82	0.34	0.37	0.69	0.88	2.15	-5.16
	(0.34, 1.94)	(0.06, 1.32)	(0.04, 2.00)	(0.13, 3.19)	(0.35, 2.17)	(0.52, 9.60)	(-13.65, 3.32)
Weight loss >15%	1.30	0.34	0.36	1.35	1.23	1.33	2.65
	(0.40, 3.88)	(0.02, 2.10)	(0.02, 2.80)	(0.23, 6.42)	(0.39, 3.90)	(0.18, 7.84)	(-8.47, 13.77)

Logistic regression models predicting long-term outcomes using sodium coefficient of variation over the first 24–96 h after birth, after adjusting for weight loss <5% 0–96 h after birth, weight loss >15% 0–96 h after birth, total fluid intake (mL/kg/d), lowest sodium <135 mEq/L within 96 h after birth, highest sodium >145 mEq/L within 96 h after birth, urine output (mL/kg/h), gestational age, sex, vasopressor use in the first week, small-for-gestational-age status, weighing, and maximum glucose (mg/dL) from the day before sodium was measured. Odds ratios with 95% CIs listed for associations. Significant associations italicized.

BPD, bronchopulmonary dysplasia; IVH, intraventricular hemorrhage; NEC, necrotizing enterocolitis; PDA, patent ductus arteriosus.

∗ Linear regression model for length of hospital stay showing the average change in number of days spent in hospital (and 95% CI) per percent change in sodium or osmolality coefficient of variation.

**TABLE 3:** Osmolality model summary for in-hospital neonatal outcomes

	Any IVH	All NEC	Surgical NEC	PDA requiring surgical treatment	BPD	In-hospital mortality	Length of hospital stay (days)∗
Osmolality coefficient of variation	0.99	1.22	1.61	1.21	1.13	0.88	-0.16
	(0.70, 1.39)	(0.75, 2.00)	(0.82, 3.25)	(0.71, 2.07)	(0.75, 1.72)	(0.57, 1.34)	(-3.69, 3.37)
Weight loss <5%	0.82	0.41	0.48	0.90	0.86	1.54	-6.02
	(0.33, 1.94)	(0.08, 1.57)	(0.06, 2.58)	(0.19, 3.76)	(0.34, 2.13)	(0.41, 5.95)	(-14.67, 2.64)
Weight loss >15%	1.10	0.27	0.27	1.24	1.17	1.76	3.56
	(0.33, 3.29)	(0.01, 1.67)	(0.01, 2.15)	(0.20, 6.09)	(0.35, 3.83)	(0.29, 8.99)	(-8.03, 15.16)

Logistic regression models predicting long-term outcomes using osmolality coefficient of variation over the first 96 h after birth, after adjusting for weight loss <5% 0–96 h after birth, weight loss >15% 0–96 h after birth, lowest osmolality <280 mEq/L within 96 h after birth, highest osmolality >300 mEq/L within 96 h after birth, total fluid intake (mL/kg/d), urine output (mL/kg/h), gestational age, sex, vasopressor use in the first week, small-for-gestational-age status, and weighing. Odds ratios with 95% CIs listed for associations. Significant associations italicized.

BPD, bronchopulmonary dysplasia; IVH, intraventricular hemorrhage; NEC, necrotizing enterocolitis; PDA, patent ductus arteriosus.

∗ Linear regression model for length of hospital stay showing the average change in number of days spent in hospital (and 95% CIs) per percent change in sodium or osmolality coefficient of variation.

### Absolute maximal sodium change compared with sodium CoV

**FIGURE 3:**
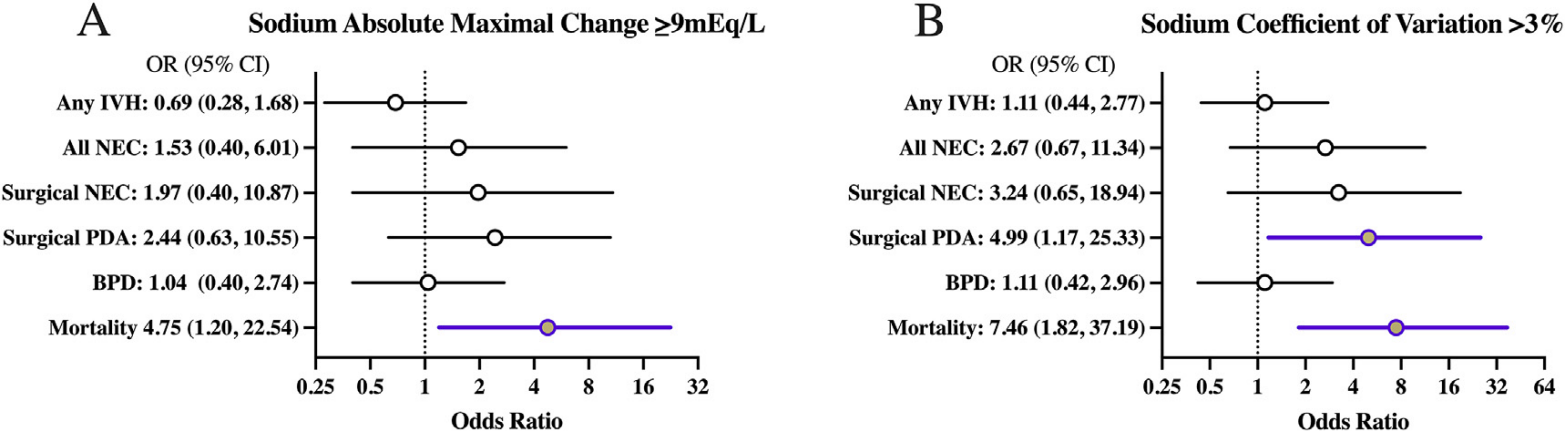
**Forest plots of serum sodium coefficient of variation or absolute maximal change and association with in-hospital neonatal outcomes**. Logistic regression model predicting long-term outcomes using sodium absolute maximal change ≥9mEq/L (A) or sodium coefficient of variation >3% (B) over the first 96 h after birth. Models were adjusted for gestational age, weighing, small-for-gestational-age (SGA) status, sex, vasopressor use in first week after birth, average urine output in first 96 h after birth (mL/kg/h), average fluid intake in first 96 h after birth (mL/kg/day), <5% weight loss 0-96 h after birth, >15% weight loss 0-96 h after birth, lowest sodium <135 mEq/L within 96 h after birth, highest sodium >145 mEq/L, within 96 h after birth, and highest glucose within 96 h after birth (mg/dL). Odds ratios with 95% CI listed for associations. Significant associations are shown in color. BPD: bronchopulmonary dysplasia, IVH: intraventricular hemorrhage, NEC: necrotizing enterocolitis, PDA: patent ductus arteriosus.

The sum of sensitivity and specificity for predicting mortality was maximized at an optimal threshold of ≥9 mEq/L for the absolute maximal sodium change and >3% for sodium CoV. At these optimal thresholds, the absolute maximal sodium change had a sensitivity of 74% and a specificity of 60%, and the sodium CoV had a sensitivity of 68% and a specificity of 70% for predicting mortality. Sodium CoV >3% was associated with higher odds of all assessed in-hospital outcomes compared with absolute maximal sodium change ≥9 mEq/L within 96 h after birth as shown in Table 4. Absolute maximal sodium change ≥9 mEq/L was associated with increased odds of overall in-hospital mortality (aOR, 4.75; 95% CI: 1.20, 22.54; Figure 3A). Sodium CoV >3% was associated with increased odds of PDA requiring surgical treatment and mortality (aOR, 4.99; 95% CI: 1.17, 25.33; aOR, 7.46; 95% CI: 1.82, 37.19, respectively; Figure 3B). As the osmolality model was not significant for any of the assessed in-hospital outcomes, further osmolality analyses were not explored.

**TABLE 4:** Comparison of sodium absolute maximal change ≥9 mEq/L and sodium coefficient of variation >3% as predictors for in-hospital outcomes

	Any IVH	All NEC	Surgical NEC	PDA requiring surgical treatment	BPD	In-hospital mortality	Length of hospital stay (d)∗
Sodium absolute maximal change ≥9 mEq/L	0.69	1.53	1.97	2.44	1.04	4.75	-3.56
	(0.28, 1.68)	(0.40, 6.01)	(0.40, 10.87)	(0.63, 10.55)	(0.40, 2.74)	(1.20, 22.54)	(-12.77, 5.64)
Sodium CoV >3%	1.11	2.67	3.24	4.99	1.11	7.46	-0.25
	(0.44, 2.77)	(0.67, 11.34)	(0.65, 18.94)	(1.17, 25.33)	(0.42, 2.96)	(1.82, 37.19)	(-9.35, 8.84)

Logistic regression models predicting long-term outcomes using sodium absolute maximal change ≥9 mEq/L or coefficient of variation >3% over the first 96 h after birth, after adjusting for weight loss <5% 0–96 h after birth, weight loss >15% 0–96 h after birth, total fluid intake (mL/kg/d), lowest sodium <135 mEq/L within 96 h after birth, highest sodium >145 mEq/L within 96 h after birth, urine output (mL/kg/h), gestational age, sex, vasopressor use in the first week, small-for-gestational-age status, weighing, and maximum glucose (mg/dL) from the day before sodium was measured. Odds ratios with 95% CIs listed for associations. Significant associations italicized.

BPD, bronchopulmonary dysplasia; CoV, coefficient of variance; IVH, intraventricular hemorrhage; NEC, necrotizing enterocolitis; PDA, patent ductus arteriosus.

∗ Linear regression model for length of hospital stay showing the average change in number of days spent in hospital (and 95% CIs) per percent change in sodium or osmolality coefficient of variation.

## Discussion

### Serum sodium and osmolality are poor proxies for weight change

Daily changes in serum sodium or osmolality within the first 4 d after birth are *poorly* associated with weight change in this study’s population. Changes in serum sodium and osmolality were only significantly associated with percent weight change in the 24-h period between days 2 and 3. Although statistically significant, these findings are unlikely to be clinically meaningful as the *R*^*2*^ was only 0.12 and 0.14 for sodium and osmolality, respectively, suggesting that changes in sodium or osmolality only predict 12% or 14% of the variation in weight. These findings may be because of the rapid reduction in total body water content and extracellular fluid over the first week after preterm birth, leading to alterations in serum electrolytes and limiting their reliability as proxies for evaluating adequate hydration [2, 11, [33], [34], [35], [36], [37], [38]]. Overall, serum electrolytes are poorly associated with weight change, and therefore are suboptimal proxies for determining fluid management decisions in the first 4 d after birth within this preterm population.

### Increasing serum sodium CoV is associated with adverse in-hospital outcomes

We have demonstrated, in this study, that the first 96 h after birth is a critical window in which increasing serum sodium variability, as measured by CoV, is associated with later development of surgical NEC and all-cause in-hospital mortality. Importantly, compared with absolute maximal sodium change, sodium CoV is a more robust measure with stronger associations with all health outcomes evaluated in our study, including overall in-hospital mortality (Table 4). Due to a paucity of data on optimal metrics evaluating sodium variability within this critical fetal-to-neonatal transition period, our study finds sodium CoV as a more accurate and meaningful measure of variability than maximal absolute change. This robust association between CoV and outcomes may be because of CoV incorporating multiple measurements over time to depict an overall trend in variability rather than a singular maximal absolute change. With premature newborns <30 wk commonly receiving frequent electrolyte evaluations over the first few days after birth, our study demonstrates CoV as a clinically meaningful and useful metric warranting further prospective evaluation.

It remains unclear whether early sodium variability is in the causative pathway or simply a consequence of critical illness. Gastrointestinal inflammation, such as with NEC, is associated with sodium fluctuations, specifically hyponatremia, but it is unknown if early fluctuations of serum sodium reflect signs of gastrointestinal inflammation or underlying injury predisposing to later development of NEC [39, 40]. Although a clear mechanism is not understood, our findings suggest increasing sodium variability in the first 96 h after birth may be an early indicator of infants at higher odds of developing NEC requiring surgical intervention or those with higher risk for all-cause in-hospital mortality. Sodium variability may also serve as a marker of inherent physiologic immaturity. Further evaluation may be best suited to basic science experiments exploring a deeper understanding of the underlying mechanism.

In contrast to sodium CoV, osmolality CoV is not significantly associated with adverse in-hospital outcomes. Thus, sodium, rather than osmolality, CoV may be a more sensitive early predictor of future adverse newborn health outcomes. The inclusion of glucose and BUN values in the calculation of osmolality may have decreased osmolality CoV’s sensitivity. Another possibility for the lack of significant relationship with in-hospital outcomes could simply be because of limitations in sample size leading to type II error.

### Future studies

Further studies are needed to evaluate whether assessing serum sodium CoV values in the first 96 h after birth provides prognostication on overall risk for surgical NEC or all-cause in-hospital mortality. Clinically, this calculation could be incorporated within electronic medical records, so it could be available to assist clinicians in their decision making. Additionally, whether daily weight change or sodium CoV is the most important for ensuring optimal newborn health outcomes is an unanswered question that deserves future research.

Overall, our study’s results add to the body of literature demonstrating the need for more intensive evaluation of the fetal-to-newborn transition period and suggest the potential benefit of using CoV to evaluate serum electrolyte variability and its association with adverse in-hospital outcomes.

### Strengths and limitations

Our study has numerous strengths. First, we included over 200 premature newborns over a contemporary 3-y period. Most previous studies exploring the relationship between fluid changes during this fetal-to-neonatal transition period included less than 20 study subjects [2, [35], [36], [37]]. These studies were commonly from the 1980s and 1990s when care included different humidification, thermoregulatory, and ventilatory strategies compared with current standard practice. Thus, our contemporary cross-sectional study design with a large sample size provides a robust evaluation of serum electrolyte changes and their relationship to adverse newborn health outcomes. Another strength included our evaluation of in-hospital outcomes beyond the first week of age. Recent literature supports the findings that fluid management and weight loss in the first 7–10 d after birth have long-lasting impact with associations for neonatal morbidity lasting for months or longer [33, 34, [41], [42], [43], [44], [45], [46]]. Our results further strengthen this growing body of knowledge by highlighting the importance of this transitional period.

Our study also had several limitations. The study was conducted within a single level IV NICU, which limits the generalizability to other settings where practice of care may differ. However, conducting the study within a single center assists in minimizing variability as the same 2 dietitians oversaw the entirety of this population to ensure limited variability in sodium administration or other aspects of care. However, as we did not collect data on specific sodium quantities administered to each patient, evaluating sodium provisions in future studies on sodium variability and newborn in-hospital outcomes is the natural next step. Generalizability may be further limited by our study’s inclusion criteria as having to survive for the first 96 h and without genetic or congenital anomalies, and the exclusion of infants with early diagnosis of IVH. Yet, we believe that including these infants would have biased our data as the most critically ill infants commonly receive high-volume resuscitation, administration of multiple medications altering glucose values including dextrose-containing fluids and steroids (and therefore osmolality or sodium via pseudohyponatremia), and other life-saving procedures altering and confounding serum electrolyte values. The adjustments within our regression models may also limit clinical applicability at this time, and we strongly recommend future, prospective studies seeking to reproduce our findings and seek understanding of clinical generalizability. Secondly, the study was retrospective and observational; we did not have an a priori sample size calculation, but will use these results to inform future, prospective studies. This study design prevents any inference of causality, and all conclusions are only associations. However, these associations provide insight into the need for more rigorous research into this transitional period and if any fluid management, nutritional, or feeding strategies can improve overall newborn health outcomes.

In conclusion, serum sodium and osmolality values were only weakly associated with weight change over the first 96 h after birth in our cohort of newborns born at ≤30 wk gestation or with ≤1250 g birth weight and, therefore, were poor proxies for weight loss. However, increasing serum sodium variability, as measured as CoV, in the first 96 h after birth was associated with greater odds of adverse in-hospital health outcomes including development of surgical NEC and all-cause in-hospital mortality in this same preterm population. Our study’s findings further highlight that in a head-to-head evaluation with absolute maximal sodium change, sodium CoV is a more robust measure of sodium variability with stronger associations with all adverse health outcomes herein studied. Ultimately, our study’s findings do not support the practice of using serum sodium or osmolality as proxies for weight loss or to determine fluid management decisions. Although preventing severe weight loss is a priority, our findings highlight the importance of tight regulation of sodium values to prevent significant variation. Further research is needed to evaluate the impact of mitigating significant fluctuations in serum sodium, as measured by CoV, in the first 96 h after birth on preterm in-hospital morbidities.

## Funding

The authors reported no funding received for this study.

## Author disclosures

The authors report no conflicts of interest.

## Data Availability

The data described in the manuscript and/or analyzed during the current study will be made available upon reasonable request from the corresponding author.
