# Supplementary material for: Increasing Sodium Variability in the First 96 Hours after Birth is Associated with Adverse In-Hospital Outcomes of Preterm Newborns
Source: Curr Dev Nutr. 2022 Dec 27;7(1):100026. doi: 10.1016/j.cdnut.2022.100026 (PMC10100926; doi:10.1016/j.cdnut.2022.100026)
Supplement: Multimedia component 1 [file mmc1.docx]

**Supplementary Data**

**Supplemental Figure 1. CONSORT Diagram.**

**
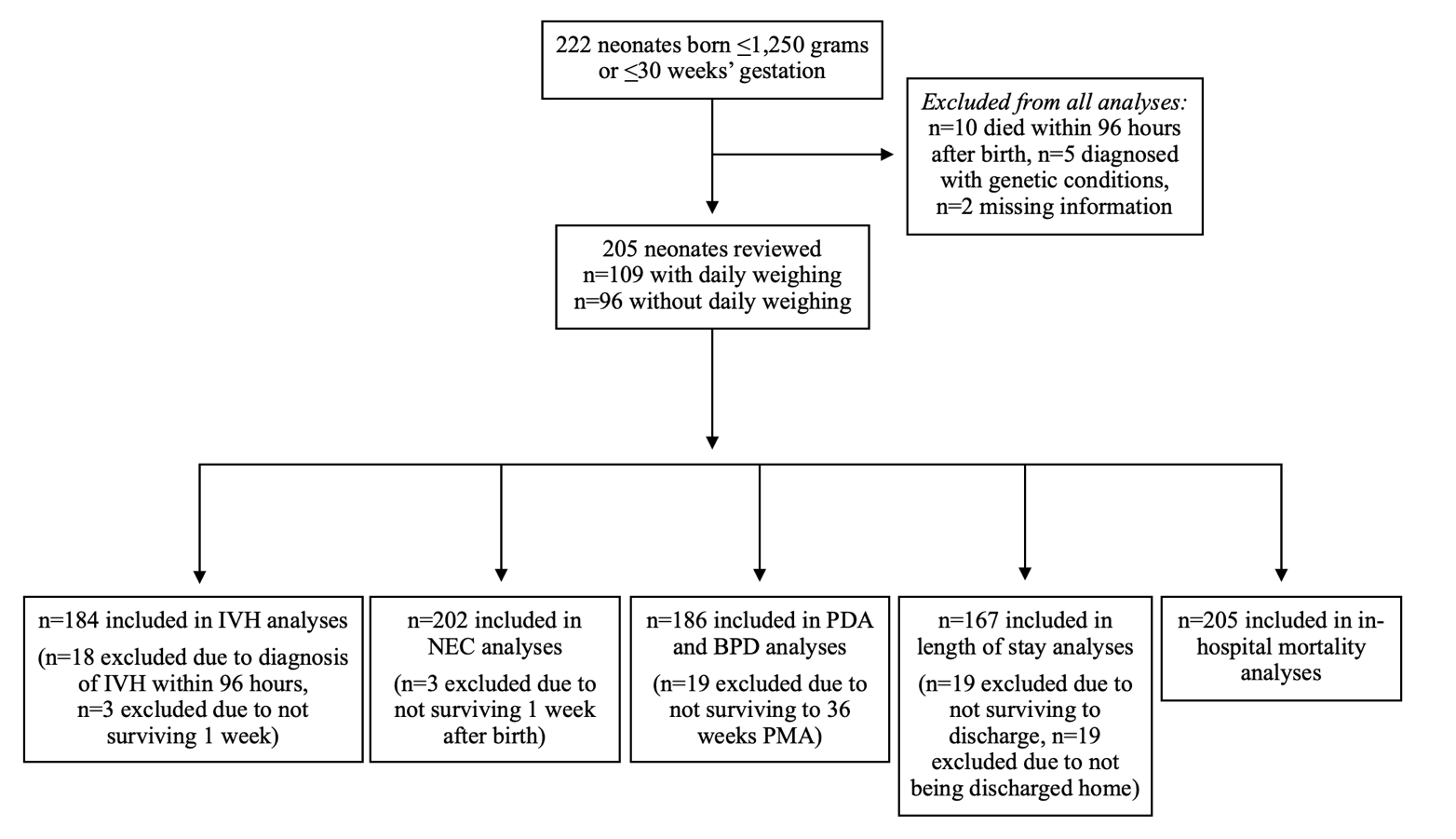
**

**Supplemental Figure 2. Coefficients of variation among infants with differing weight loss 0-96 hours after birth.**


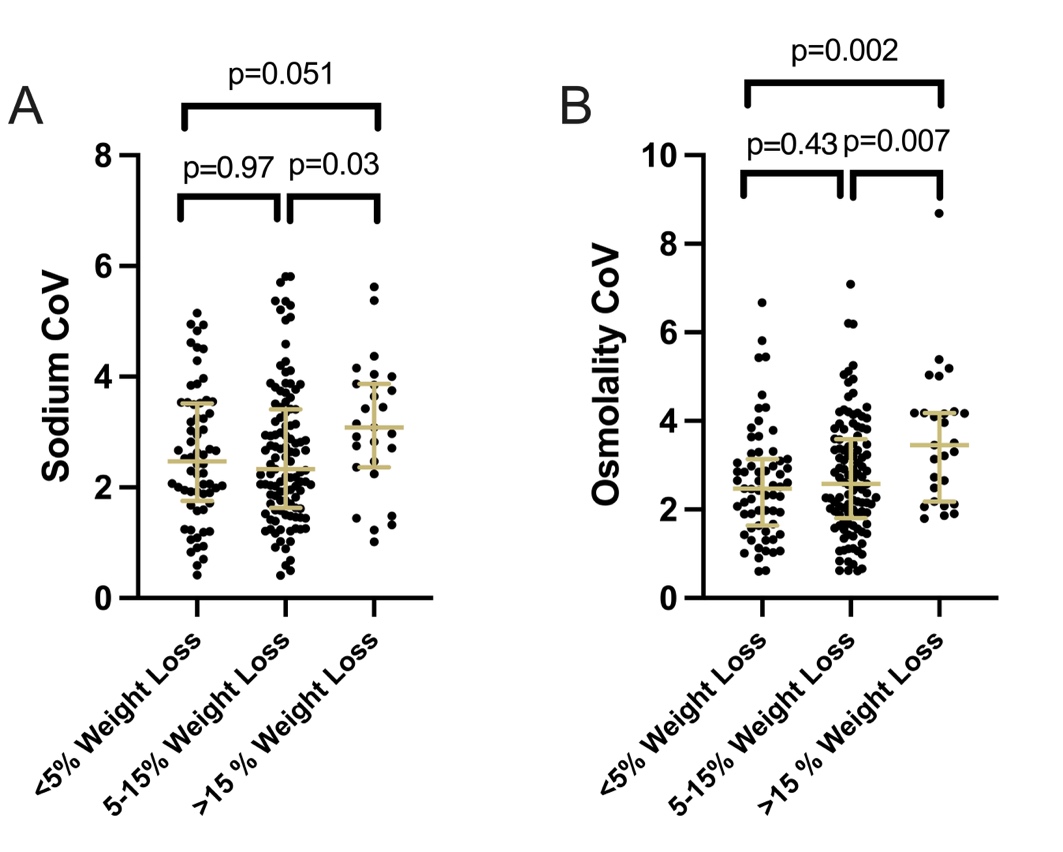


Sodium coefficient of variation (A) and osmolality coefficient of variation (B) among infants with <5%, 5-15%, and >15% weight loss 0-96 hours after birth. Mann Whitney U-tests revealed, on average, infants with 5-15% weight loss had lower sodium CoV than infants with >15% weight loss (p=0.03). There was no significant difference in sodium CoV between infants with <5% weight loss and 5-15% weight loss (p=0.97), or <5% weight loss and >15% weight loss (p=0.051). On average, infants with <5% weight loss had a lower osmolality CoV than infants with >15% weight loss (p=0.002), and infants with 5-15% weight loss had a smaller osmolality CoV than infants with >15% weight loss (p=0.007). There was no significant difference in osmolality CoV between infants with <5% weight loss and 5-15% weight loss (p=0.43).
